# The Role of Low-Energy Electron Interactions in cis-Pt(CO)_2_Br_2_ Fragmentation

**DOI:** 10.3390/ijms22168984

**Published:** 2021-08-20

**Authors:** Maicol Cipriani, Styrmir Svavarsson, Filipe Ferreira da Silva, Hang Lu, Lisa McElwee-White, Oddur Ingólfsson

**Affiliations:** 1 Department of Chemistry and Science Institute, University of Iceland, Dunhagi 3, 107 Reykjavik, Iceland; mac31@hi.is (M.C.); styrmir94@gmail.com (S.S.); 2 CEFITEC, Departamento de Física, Faculdade de Ciências e Tecnologia, Universidade NOVA de Lisboa, 2829-516 Caparica, Portugal; fr.silva@fct.unl.pt; 3 Department of Chemistry, University of Florida, Gainesville, FL 32611-7200, USA; hlu90@ufl.edu (H.L.); lmwhite@chem.ufl.edu (L.M.-W.)

**Keywords:** anticancer drugs, cisplatin, platinum (II) halo-carbonyl complexes, low-energy electrons, dissociative electron attachment, dissociative ionization, focused electron beam induced deposition

## Abstract

Platinum coordination complexes have found wide applications as chemotherapeutic anticancer drugs in synchronous combination with radiation (chemoradiation) as well as precursors in focused electron beam induced deposition (FEBID) for nano-scale fabrication. In both applications, low-energy electrons (LEE) play an important role with regard to the fragmentation pathways. In the former case, the high-energy radiation applied creates an abundance of reactive photo- and secondary electrons that determine the reaction paths of the respective radiation sensitizers. In the latter case, low-energy secondary electrons determine the deposition chemistry. In this contribution, we present a combined experimental and theoretical study on the role of LEE interactions in the fragmentation of the Pt(II) coordination compound cis-PtBr_2_(CO)_2_. We discuss our results in conjunction with the widely used cancer therapeutic Pt(II) coordination compound cis-Pt(NH_3_)_2_Cl_2_ (cisplatin) and the carbonyl analog Pt(CO)_2_Cl_2_, and we show that efficient CO loss through dissociative electron attachment dominates the reactivity of these carbonyl complexes with low-energy electrons, while halogen loss through DEA dominates the reactivity of cis-Pt(NH_3_)_2_Cl_2_.

## 1. Introduction

Platinum coordination complexes, such as cisplatin [Pt(NH_3_)_2_Cl_2_], have been widely used as chemotherapeutic anticancer drugs since the 1970s [1,2]. Cisplatin-based chemotherapy has proven to be highly effective against testicular cancer [3,4] and other various types of malignancies, such as metastatic melanoma, carcinoma of the head and neck and metastatic breast cancer [5]. The cytotoxicity of this platinum complex lies in its ability to form CDDP–DNA adducts inhibiting replication [1,2]. In fact, after entering the cell, the cisplatin undergoes hydrolysis, and as a result, the two chlorine atoms are lost. The remaining fragment forms the CDDP–DNA adducts by binding to guanine or purine nucleobases that inhibit transcriptions of the cancer cell, blocking its replication. The synchronous combination of platinum complexes and radiation (chemoradiation) has increased the survival probability of cancer patients due to the enhancement of the cell-killing effect of radiation [6,7,8]. It has been hypothesized that cisplatin, in addition to acting directly as a chemotherapeutic agent, also acts as a radiosensitizer. Apart from the medical/biological applications, platinum-based complexes are also used as precursors for focused electron beam-induced deposition (FEBID) [9,10,11,12,13], an electron-induced materials deposition technique for the fabrication of functional nanostructures [14]. In both applications, low-energy electrons (LEEs) play an important role in dictating the fragmentation pathways through electron capture as well as by electron ionization. In chemoradiation, these low-energy electrons are produced in the interaction of the ionizing radiation with the tissue material and in FEBID by interaction of the high energy electron beam with the substrate surface and the depositing material [15,16]. In both cases, the energy distribution of these secondary electrons peaks at or below 10 eV, and has a contribution close to 0 eV and a long tail extending to higher energies [17]. These LEEs can inflict considerable damage on the DNA [18], causing single and double strand breaks (SSB and DSB) [19]. In a 2008 study by Zheng et al. [7], the authors showed that when cisplatin is covalently bonded to DNA, SSB and DSB induced by LEEs are substantially enhanced. This enhancement has been attributed to bond cleavage triggered by the formation of transient negative ions (TNI) through electron capture, i.e., dissociative electron attachment (DEA). Dissociative electron attachment studies on cisplatin have been performed by Kopyra et al. [20], determining the fragmentation pathways under interaction with low-energy electrons. In this study, it was shown that electrons close to 0 eV can easily fragment this molecule by cleavage of the Pt−Cl bonds, leading to the loss of one or even both Cl atoms with considerable intensity. Hence, one single low-energy electron efficiently triggers the cleavage of both the Pt−Cl bonds. In the FEBID process, gas phase precursors, usually organometallics, are introduced into a high vacuum (HV) chamber in close proximity to a substrate surface where they are subjected to a tightly focused high-energy electron-beam. The high energy electron beam generates a considerable number of low-energy SEs [21,22] that interact with the precursor molecules, initiating chemical reactions through DEA, dissociative ionization (DI), neutral dissociation (ND) and dipolar dissociation (DD). These processes determine the decomposition of the precursor molecule at the substrate surface leading to deposition of the nonvolatile fragments formed, while volatile fragments are pumped away. A description of these processes is given, e.g., in References [23,24]. Ideally, for the creation of metallic deposits, the organometallic precursors should fully dissociate, leaving the metallic atom on the surface while the ligands are pumped away. In recent years, several studies on LEE interactions with organometallic platinum compounds have been carried out in the context of FEBID [25,26,27,28], studies that are also relevant to the action of radiation sensitizers where low-energy electrons are expected to play a significant role.

In low-energy electron interactions with Pt(CO)_2_Cl_2_ in the gas phase under single collision conditions, Ferreira da Silva et al. [28] found that, in contrast to cisplatin [20], DEA close to 0 eV electron incident energies leads exclusively to CO loss. This channel is very efficient for both single and double CO loss, while Cl loss is inefficient and only observed at higher energies. Similarly, CO loss is also the main channel in dissociative ionization of this compound, though significant Cl loss is also observed and the bare Pt^+^ ion is formed with appreciable intensity. In a UHV surface study where adsorbed monolayers of Pt(CO)_2_Cl_2_ (and Pt(CO)_2_Br_2_) were exposed to 500 eV electrons from a flood gun (and correspondingly the generated low-energy SEs), J. A. Spencer et al. [12] found that these compounds decomposed by rapid CO loss leading to a PtCl_2_ deposit with the 1:2 stoichiometric ratio of the initial compound. Prolonged electron irradiation then led to nearly quantitative removal of the chlorine. The authors attributed this to an initial DEA step leading to the CO loss. In this context, a comparative electron beam deposition study of Pt(CO)_2_Cl_2_ and Pt(CO)_2_Br_2_ was recently conducted by A. Mahgoub et al. [29]. Interestingly, it was found that while both these compounds behave similarly, the UHV deposits contained a significant portion of the halogen species but little or no carbon, while the deposits created under HV contained only small amounts of halogen species but high carbon content. It is possible that the presence of trace water in the HV experiments leads to the formation of volatile HCl in the irradiation process, decreasing the chlorine content in the deposits. This has been observed by M. Rohdenburg et al. [13,30] for cisplatin in electron-induced intermolecular reactions of the chlorine with hydrogen from the amine ligand and in electron-induced reactions of (η^3^-C_3_H_5_) Ru(CO)_3_Cl in the presence of NH_3_ as processing gas. It is thus clear that the environment plays a critical role in electron-induced decomposition of these compounds, and this is especially true for biological media where water is plentiful.

In the current study, we have performed DEA and DI experiments on Pt(CO)_2_Br_2_ in the gas phase under single collision conditions as well as thermochemical calculations at the DFT and coupled-cluster level of theory for the respective processes. Relaxed potential energy surface scans were computed, and vertical electron attachment energies and the respective MOs were calculated along with the respective electronic excitation energies. For comparison, the vertical transition energies from the anionic ground state to the first excited anionic state are also presented for all the Pt(II) halogen carbonyls; Pt(CO)_2_X (X = F, Cl, Br and I). We compare our findings with previous work on electron-induced decomposition of Pt(CO)_2_Cl_2_ and of Pt(CO)_2_Br_2_ as well as cisplatin; Pt(NH_3_)_2_Cl_2_.

## 2. Results and Discussion

**Figure 1:**
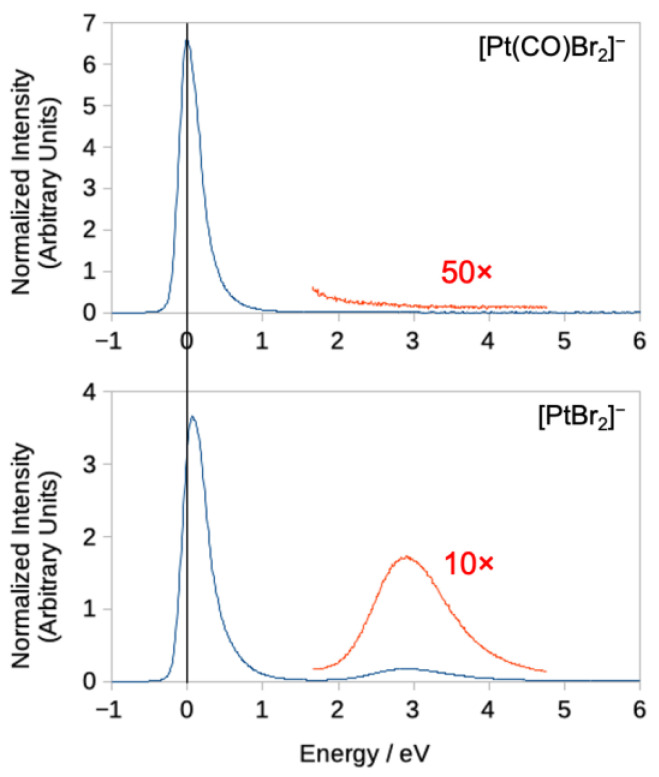
Negative ion yields for the loss of one and two CO ligands. **Top**: loss of one CO ligand; **bottom**: loss of two CO ligands.

Figure 1 shows the DEA ion yield curves for Pt(CO)Br_2_^−^ and PtBr_2_^−^, from Pt(CO)_2_Br_2_, i.e., the energy dependence of the loss of one and two CO ligands, respectively. To better allow comparison, the intensities are normalized with respect to the pressure and the intensity of SF_6_^−^ formation from SF_6_ at 0 eV recorded before each measurement. These are the two most efficient DEA processes and both fragments are formed with appreciable intensity close to 0 eV. However, while [Pt(CO)Br_2_]^−^ peaks at 0 eV, the maximum of the low-energy PtBr_2_^−^ contribution is at about 0.07 eV, and both contributions are broad and asymmetric towards higher energies. The loss of both CO ligands, and the formation of PtBr_2_^−^, is also observed through a higher-lying resonance (or resonances), contributing to the ion yield close to 3 eV. This contribution is not observed in the single CO loss ion yield curve. This is similar to the previous observations for Pt(CO)_2_Cl_2_ [28], where DEA contributions through higher lying resonances were observed in the [PtCl_2_]^−^ but not in the [Pt(CO)Cl_2_]^−^ ion yield curves. The single CO loss from Pt(CO)_2_Cl_2_ was found to be exothermic, and it was suggested that the excess energy in the [Pt(CO)Cl_2_]^−^ fragment (i.e., after the first CO loss) makes its survival probability low at the onset of the high energy resonance (or resonances), and further decomposition to [PtCl_2_]^−^ is the predominant process. The losses of one and two CO ligands from Pt(CO)_2_Br_2_ through DEA are also found to be exothermic, and at the DLPNO-CCSD(T) level of theory, we find the threshold for the loss of one and two CO ligands to be −1.57 and −0.48 eV, respectively.

**Figure 2:**
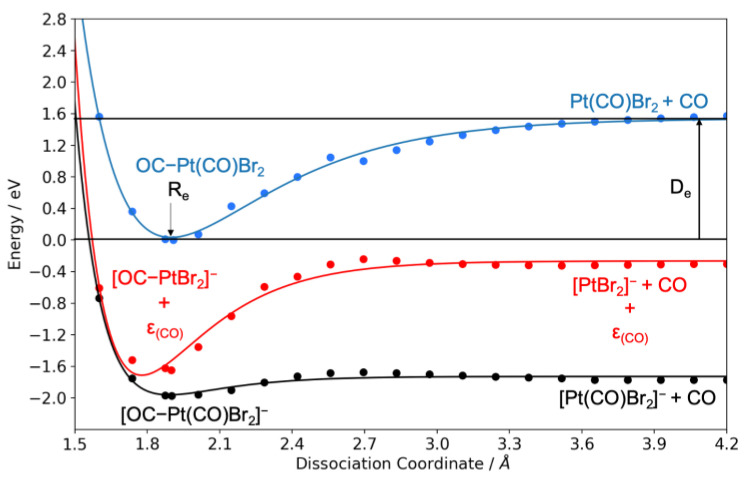
Relaxed potential energy surface scans for OC−Pt(CO)Br_2_ dissociation from the neutral parent and OC−Pt(CO)Br_2_ and OC−PtBr_2_ dissociations from the respective anion. The calculations were performed at wB97X-D3/ma-def2-TZVP level of theory.

This is also clear from the cut through the relative potential energy surfaces (PESs) shown in Figure 2. These are calculated through relaxed energy scans at the wB97X-D3/ma-def2-TZVP level of theory along the OC−Pt(CO)Br_2_ and OC−PtBr_2_ dissociation coordinates, respectively. For [Pt(CO)Br_2_]^−^, the energy contribution of the CO ligand (ε_(CO)_) is included in the calculations. The single-point energies obtained from the relaxed energy scans were fitted with Morse potential energy function. From the fitting, we obtained the Pt−CO dissociation energies from the minimum of the potential curve (D_e_) and the Pt−CO bond lengths (R_e_) for the neutral molecule (Pt(CO)_2_Br_2_), the molecular anion ([Pt(CO)_2_Br_2_]^−^), and the [Pt(CO)Br_2_]^−^ fragment. It is noted that these PESs do not include the zero-point vibrational energy (ZPVE). For the neutral molecule, we derived a dissociation energy of 1.5 eV and an equilibrium Pt–CO bond length of 1.9 Å. For the molecular anion, these values were found to be 0.2 eV and 1.9 Å, respectively. The Pt−CO bond length in [Pt(CO)Br_2_]^−^ was found to be 1.8 Å and the dissociation energy was found to be 1.4 eV. The corresponding Pt−CO bond lengths from our geometry optimization at the wB97X-D3/ma-def2-TZVP level of theory are 1.9 Å for Pt(CO)_2_Br_2_, [Pt(CO)_2_Br_2_]^−^ and [Pt(CO)Br_2_]^−^, which agrees with these derived from the PES fits.

As is clear from Figure 2, the PESs for the formation of the anionic fragment [Pt(CO)Br_2_]^−^ and [PtBr_2_]^−^ lie entirely below the ground state of the neutral molecule in the range of the dissociative coordinate 1.4 to 4.2 Å. Thus, the survival probability of [Pt(CO)Br_2_]^−^, with respect to further CO loss, drops rapidly above the threshold. This, in turn, is reflected in the shift and broadening of the low-energy contribution for [Pt(CO)Br_2_]^−^ as compared to PtBr_2_^−^ and the lack of any [Pt(CO)Br_2_]^−^ contribution through the higher lying resonance at around 3 eV.

**Figure 3:**
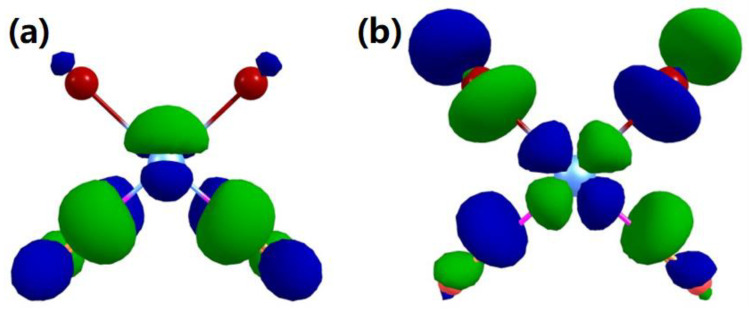
Isosurfaces of the frontier orbitals of the Pt(CO)_2_Br_2_ anion obtained with a contour value of 0.05. (**a**): SOMO. (**b**): SOMO+1.

Similar to Pt(CO)_2_Cl_2_ [28], we attribute the low-energy contributions in the Pt(CO)_2_Br_2_ ion yields to the initial formation of the ground state negative ion and the first excited negative ion state, i.e., electron occupation of the LUMO and the slightly higher lying LUMO+1. Figure 3 shows the electrostatic potential isosurfaces for the corresponding SOMO and SOMO+1. The former of these is a mixture of contributions from the π* CO orbitals and the Pt 5 d_xz_ p and has antibonding character. The latter is predominantly composed of the Pt $d_{x^{2}-y^{2}}$ orbital with σ* P−L antibonding character (L = CO or Br) and direct CO loss from this excited anion state is given by its repulsive σ* nature. The same process from the ground anion state, however, is in principle symmetry forbidden and requires effective coupling of the CO π* orbital with the respective σ* Pt−L. For Pt(CO)_2_Cl_2_ [28], it has been hypothesized that such effective coupling is provided by the out-of-plane bending of the CO group.

From our calculation, we derive a dipole moment for Pt(CO)_2_Br_2_ of 5.0 D, which should be well above the limit for a capture into a dipole bound state [31]. For the lower lying (0 eV) resonance, we anticipate that this provides a gateway for the DEA process as has been discussed by, e.g., Sommerfeld for nitromethane, uracil and cyanoacetylene [32]. In this mechanism, the initially formed diffuse dipole-bound state couples with the nuclear motion, channeling the excess energy into the vibrational degrees of freedom. The so formed vibrational Feshbach resonance couples with the respective valence state, in this case the LUMO, leading to a transient negative ion characterized by the excess electron defining the SOMO. At higher energies, where the angular momentum of the electron has *l* components higher than zero, the initial capture may rather be through the formation of the respective shape resonance. A detailed discussion on the actual capture mechanism at these low incident energies exceeds the scope of this paper, but we refer the interested reader to Reference [24] and references therein.

The calculated adiabatic electron affinity of Pt(CO)_2_Br_2_ was found to be 2.31 eV at the ⍵B97X-D3 level of theory and 2.11 eV at the DLPNO-CCSD(T)/aug- cc-pVQZ level of theory. The vertical attachment energy (VAE) for the anion ground state was found to be −1.14 eV at the ⍵B97X-D3 level of theory and −0.99 eV for the first excited anion state using a delta-SCF approach at the same level of theory. Similar to PtCl_2_(CO)_2_, the VAEs associated with formation of the ground state and first excited state anions are both negative at the equilibrium geometry of the neutral molecule. The resulting vertical excitation energy for PtBr_2_(CO)_2_ from the anion ground state to the first anionic excited state was found to be 0.15 eV, while for PtCl_2_(CO)_2_, the value was found to be 0.38 eV with delta-SCF at the same level of theory [28]. This reflects the increased destabilization of the σ* SOMO+1 with increasing electronegativity of the halogen ligand and suggests that substitution of the Br ligands with I atoms would further decrease the excitation energy while substitution with F atoms would largely increase the excitation energy. This is confirmed by the vertical transition energies between these anion states, obtained at TDDFT wB97X-D3/ma-def2-TZVP level of theory, shown in Table 1. As can be seen in Table 1, the vertical excitation energy from the anionic ground state to the first anionic excited state decreases following the trend [Pt(CO)_2_F_2_]^−^ > [Pt(CO)_2_Cl_2_]^−^ > [Pt(CO)_2_Br_2_]^−^ > [Pt(CO)_2_I_2_]^−^.

**Figure 4:**
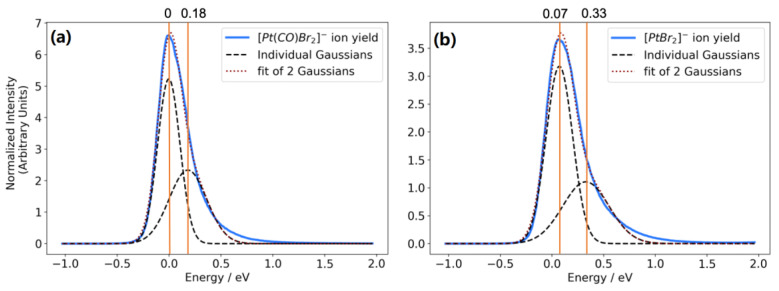
Fit of two Gaussian functions to the [Pt(CO)Br_2_]^−^ ion yield (**a**) and the [PtBr_2_]^−^ ion yield (**b**). The first represents contributions from dissociation from the anionic ground state, while the latter represents these from the first excited anionic state.

This is also reflected in the more structured low-energy contributions from Pt(CO)_2_Cl_2_ as compared to the high energy tail observed in Pt(CO)_2_Br_2_. To visualize this, Figure 4 shows a fit of two Gaussian functions to the low-energy contributions in the ion yield curves for [Pt(CO)Br_2_]^−^ and PtBr_2_^−^. An excellent fit with an R^2^ value of 0.99 is achieved with a fairly narrow lower energy contribution peaking at about 0 eV electron energy and a broader higher energy component peaking at 0.18 and 0.33 eV, respectively. These values for the higher energy contributions are in both cases slightly below the corresponding VAE as is to be expected due to the intrinsic competition between autodetachment and dissociation. The lower value for [Pt(CO)Br_2_]^−^ as compared to [PtBr_2_]^−^ is also in line with the expected energy dependence of the survival probability of that fragment with respect to further CO loss to form [PtBr_2_]^−^.

**Figure 5:**
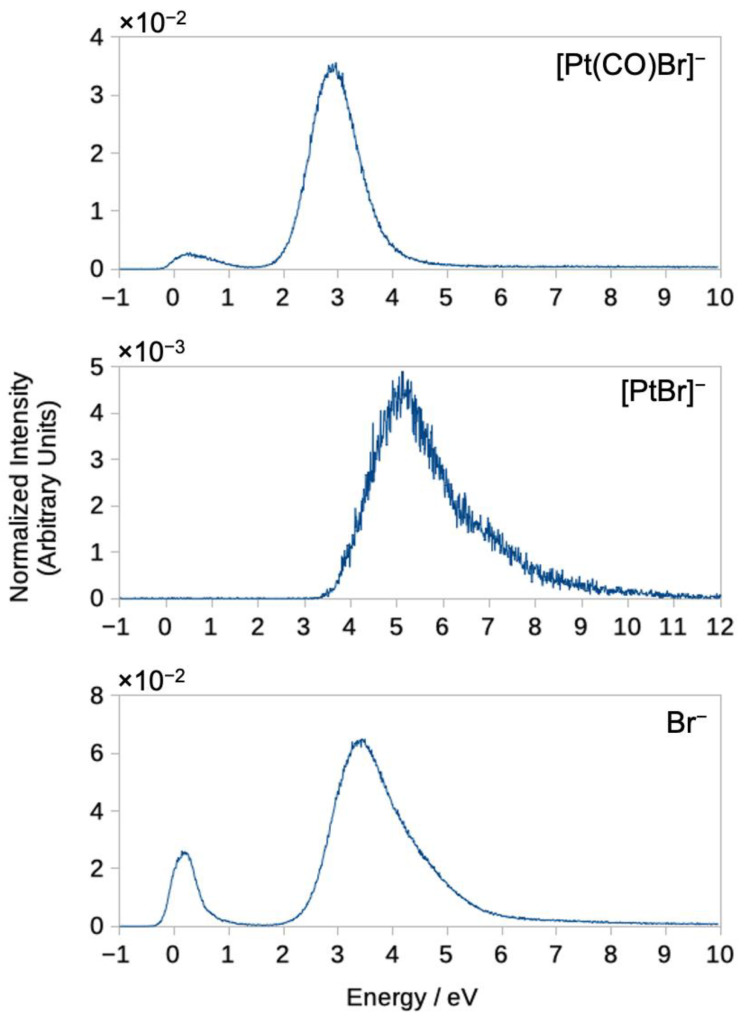
Negative ion yields for [Pt(CO)Br]^−^, [PtBr]^−^ and Br^−^.

In addition to the CO loss fragments, DEA to Pt(CO)_2_Br_2_ also leads to the formation of [Pt(CO)Br]^−^, [PtBr]^−^ and Br^−^, though with considerably lower intensity. The ion yield traces for these are shown in Figure 5, and Table 2 lists the threshold values for all fragments observed from Pt(CO)_2_Br_2_, calculated at the wB97X-D3/ma-def2-TZVP and DLPNO-CCSD(T)/aug-cc-pVQZ level of theory. For comparison, the onsets of individual contributions estimated from the ion yield curves, i.e., the appearance energies (AEs), are also shown in Table 2. With the exception of the formation of [PtBr]^−^, all DEA channels observed from Pt(CO)_2_Br_2_ are found to be exothermic. Similar to [PtBr_2_]^−^, the ion yield curve for [Pt(CO)Br]^−^ shows two contributions, one that peaks close to 0.5 eV and one with considerably higher intensity peaking close to 3 eV. We attribute the former of these to dissociation from the first excited anionic state, though contributions from the high energy tail from the ground state transient negative ion (TNI) cannot be excluded. The 3 eV contribution is shifted to slightly higher energies as compared to the double CO loss, which is likely rooted in the competition between these channels falling in favor of the more exothermic double CO loss at lower energies. The situation is very similar for the Br^−^ formation, which also appears through two contributions peaking at around 0.15 and 3.4 eV, respectively, and here we also attribute the low-energy contribution to the σ*, first anionic excited state. Interestingly we do not observe any [Pt(CO)_2_Br]^−^ contributions in DEA to Pt(CO)_2_Br_2_, suggesting that [Pt(CO)Br]^−^ is formed through initial CO loss, i.e., through Br loss from [Pt(CO)Br_2_]^−^. This may be rooted in the synergistic back-bonding Br−Pt−CO interaction through the contribution of Br lone pair electron density through the metal d orbitals into the π*(CO). If the CO is lost first, then the partial extra bond from Br−Pt is gone, and the Br that was trans to the now missing CO may be more disposed to dissociation.

Finally, [PtBr]^−^ is formed with an onset close to its threshold at 3.8 eV and a maximum at about 5 eV. This fragment, which is formed through the loss of three ligands, appears with low intensity, and we anticipate that it is formed through the high energy tail of the resonance (or resonances), contributing to the [PtBr_2_]^−^, [Pt(CO)Br]^−^ and Br^−^ formation at around 3 eV.

At the wB97X-D3/ma-def2-TZVP level of theory, taking into account the ZPVE and the thermal energy corrections at room temperature, we find the halogen−Pt bond energies for Pt(CO)_2_Cl_2_ and Pt(CO)_2_Br_2_ to be 3.3 and 2.8 eV, respectively. The calculated threshold for the formation of [Pt(CO)Br]^−^ was found to be −0.017 eV at the DLPNO-CCSD(T) level of theory, while the threshold for [Pt(CO)Cl]^−^ was found to be 0.19 eV at the same level of theory. Similarly, the calculated threshold for the formation of Br^–^ was found to be −0.59 eV at the DLPNO-CCSD(T) level of theory, while the calculated threshold for Cl^−^ formation from Pt(CO)_2_Cl_2_ was found to be −0.51 eV at the same level of theory. It is noted that the [Pt(CO)Br]^−^ calculations include the thermal energy of the neutral at 50 °C, while those for Pt(CO)_2_Cl_2_ include the thermal energy of the neutral at 85 °C.

As compared to Pt(CO)_2_Cl_2_ and Pt(CO)_2_Br_2_, DEA to cisplatin [Pt(NH_3_)_2_Cl_2_] shows a very different behavior. Here, the dominant DEA channels are the formation of [Pt(NH_3_)_2_Cl]^−^, Cl^−^ and [Pt(NH_3_)_2_]^−^ [20], i.e., the cleavage of the Pt–halogen bonds. This is interesting, as similar to the Pt−CO bond, the Pt−NH_3_ bond is much weaker than the Pt−Cl bond, and in their study, Kopyra et al. [20] calculated the respective bond energies to be 1.5 eV for the Pt–NH_3_ bond and 3.3 eV for the Pt−Cl bond using DFT. The LUMO, involved in LEE attachment to cisplatin, however, has a repulsive σ* character in all the ligands [20], which is more similar to the LUMO+1 of the current compound, through which effective Pt−Br bond cleavage is observed. Thus, at very low energies, where the electron attachment cross sections are the highest, direct relaxation of [Pt(NH_3_)_2_Cl_2_]^−^ by lengthening of the Pt−Cl bond beyond its bonding distance is allowed while this process is in principle symmetry forbidden from the π*_CO_ character LUMO of [Pt(CO)_2_Cl]^−^ and [Pt(CO)_2_Br]^−^.

**Figure 6:**
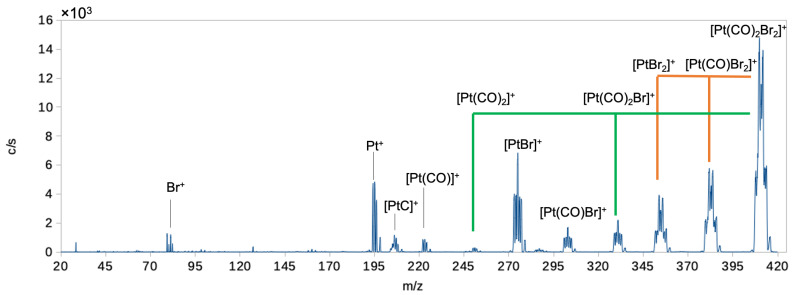
Positive ion mass spectrum of Pt(CO)_2_Br_2_ recorded at 70 eV incident electron energy. Green lines show the sequential loss of the two Br ligands, while orange lines show the sequential loss of the two CO ligands.

Dissociative ionization of Pt(CO)_2_Br_2_ leads to much more extensive fragmentation than DEA. Figure 6 shows the positive ion DI spectrum of Pt(CO)_2_Br_2_ recorded at 70 eV electron energy, and Table 3 lists the relative contributions of individual fragments normalized to the contribution of the parent ion as well as the efficiency of CO and Br removal per incident electron. The DI spectrum shows all the fragments associated with the breakdown of the Pt(CO)_2_Br_2_. As for Pt(CO)_2_Cl_2_, the dominant contribution is from the parent ion [Pt(CO)_2_Br_2_]^+^, and the bare Pt^+^ ion is observed with significant intensity. The loss of one and two CO, i.e., the formation of [Pt(CO)Br_2_]^+^ and [PtBr_2_]^+^, is also appreciable, while the loss of one Br and two Br, i.e., [Pt(CO)_2_Br]^+^ and [Pt(CO)_2_]^+^, is much less significant. The formation of [PtBr]^+^ is appreciable, while other fragments appear with marginal intensity.

Finally, for comparison with the electron-induced decomposition of Pt(CO)_2_Br_2_ in FEBID and at surfaces, we have calculated the average bromine and carbonyl loss per incident in DEA and DI (see Table 3). The average CO loss per incident in DEA was calculated by taking the integral intensities of all ion yield curves of CO-loss fragments from about 0 to 10 eV (see Figure 1) and weighing these by the number of carbonyls lost. In a similar way, the average Br loss through DEA was obtained by weighing the integral intensities of the [Pt(CO)Br]^−^, [PtBr]^−^ and Br^−^ ion yield curves by the number by bromines lost. For DI, the average CO and Br losses per incident were obtained in a similar way by integrating over the isotope distribution of the respective fragment peaks in the positive ion mass spectrum and weighing these by the number of CO and Br lost in the respective processes. In DEA we found an average CO loss of 1.4 per incident through DEA, and an average Br loss per incident of only 0.04. For DI, we found an average CO loss of 0.7 per incident and an average Br loss of 0.3. This is comparable to the observations made for Pt(CO)_2_Cl_2_ [28], where the average CO loss in DEA is also found to be 1.4 and the Br loss negligible, while DI leads to an average of 0.6 CO and 0.5 Br lost per incident, respectively. In electron-induced decomposition of Pt(CO)_2_Cl_2_ at surfaces [12], CO loss was found to be the dominating process at low electron doses, leading to an average CO loss of around 1–2. Similarly, in UHV electron-induced deposition experiments, the deposits made with Pt(CO)_2_Br_2_ and Pt(CO)_2_Cl_2_ were found to retain the nearly 1:2 platinum:halogen ratio of the precursor compounds [29]. These surface and deposition studies suggest that DEA rather than DI is dominating in the initial decomposition step. However, as mentioned in the introduction, depositions made with Pt(CO)_2_Cl_2_ and Pt(CO)_2_Br_2_ under HV conditions were found to contain very little of the respective halogen species with carbon being the main contaminant [29].

For electron irradiation of cisplatin at surfaces [13], it has been shown that intermolecular reactions of the chlorine with hydrogen from the amino ligands readily produce HCl that desorbs and effectively reduces the Cl content in the adsorbate. Similarly, the use of NH_3_ as a processing gas in electron-induced decomposition of (η^3^-C_3_H_5_)Ru(CO)_3_Cl at surfaces has proven effective in Cl removal through HCl formation [30]. We speculate that residual water may have the same effect in FEBID of Pt(CO)_2_Cl_2_ and Pt(CO)_2_Cl_2_ under HV conditions and thus explain the very different observations under HV and UHV conditions. This is important when considering electron-induced decomposition in a biological environment, where water is omnipresent.

## 3. Materials and Methods

### 3.1. Experimental Setup

Low-energy electron interactions with Pt(CO)_2_Br_2_ were studied in a crossed electron-molecule beam apparatus. The experimental setup has been covered previously [33], and only a short description will be given here. The instrument consists of a quadrupole mass spectrometer (HIDEN EPIC1000), a trochoidal electron monochromator (TEM) and an effusive gas inlet system. The TEM and the ion extraction elements are maintained at 120 °C during measurements to avoid target gas condensation on the electrical lens components. A quasi mono-energetic electron beam, generated with the TEM, crosses an effused molecular beam of the target gas. The ions formed in the crossed beam region are then extracted from the reaction region by a weak electric field (<1 V/cm) and analyzed by the mass spectrometer. Both positive and negative ions can be studied, and ion yield curves are recorded by scanning through the electron energy at a fixed mass (*m*/*z*), while mass spectra are recorded by scanning through the relevant *m*/*z* range at fixed energies.

The background pressure inside the chamber is on the order of 10^−6^ Pa and the pressure during measurements typically about 10^−5^ Pa to ensure single collision conditions. The energy scale was calibrated by the well documented SF_6_^−^ formation from SF_6_ at 0 eV [34] recorded before and after each measurement. The energy resolution was estimated from the FWHM of that signal and was found to be 100–150 meV for the current measurements. PtBr_2_(CO)_2_ is solid at room temperature and was sublimed at ~50 °C in the gas inlet system.

### 3.2. PtBr_2_(CO)_2_ Synthesis

PtBr_2_(CO)_2_ was synthesized at the University of Florida as previously reported [29] and characterized by comparison to literature data [35].

### 3.3. Computational Method

Ab initio calculations were performed with the quantum chemistry package ORCA 4.2.1 [36]. All geometries were first optimized using a range-separated hybrid functional ωB97X-D3 [37,38] with minimal augmented triple zeta basis set, ma-def2-TZVP [39,40] and the def2 effective core potential (ECP) [41] for platinum core electrons. Harmonic vibrational frequencies of the molecule and fragments were calculated at the same level of theory. They were confirmed to be positive, i.e., all structures were stationary points on the potential energy surface, and were used to yield zero-point energy contributions for the molecule and all the fragments as well as thermal energy correction for the neutral parent at 50 °C. Final threshold energies were calculated using the coupled cluster approach at the DLPNO-CCSD(T) [42,43,44,45] level of theory on the ⍵B97X-D3 optimized geometries. A large and diffuse aug-cc-pVQZ basis set [46,47,48] (aug-cc-pVQZ-PP basis set and associated pseudopotential for Pt) [49] was used. All thresholds include zero-point vibrational energies for all fragments and the thermal energy of the neutral molecule at 50 °C (⍵B97X-D3/ma-def2-TZVP level of theory). Vertical excitation energies for the first excited anion states were calculated using time-dependent DFT (TDDFT) at the ⍵B97X-D3/ma-def2-TZVP level of theory.

## 4. Conclusions

Dissociative electron attachment to Pt(CO)_2_Br_2_ in the energy range from about 0 to 12 eV and dissociative ionization at 70 eV were studied in a crossed electron-molecule beam experiment. The thermochemical thresholds for all DEA channels were calculated, and relaxed potential energy surface scans were computed for the main channels. The vertical electron attachment energies and the respective MOs were calculated for the lowest lying anionic states as well as the vertical transition energy from the anionic ground state to the first excited anionic state for the Pt(II) halogen carbonyls Pt(CO)_2_X (X = F, Cl, Br and I). In DI at 70 eV, the main contributions are from the parent ion [Pt(CO)_2_Br_2_]^+^, but the loss of one and two CO, [Pt(CO)Br_2_]^+^ and [PtBr_2_]^+^, and the formation of PtBr^+^ and the bare Pt^+^ ion are also significant. The loss of one and two Br, [Pt(CO)_2_Br]^+^ and [Pt(CO)_2_]^+^, the formation of [Pt(CO)Br]^+^, and the platinum carbide PtC^+^ and Br^+^ are also apparent, though with lesser intensities. All DEA channels, except the formation of PtBr^−^ were found to be exothermic, and the dominating DEA channels are the loss of one and two CO leading to the formation of [Pt(CO)Br_2_]^−^ and [PtBr_2_]^−^, while Br loss is insignificant. The CO loss appears predominantly through fairly broad contributions in the respective ion yields, peaking close to 0 eV and markedly asymmetric towards higher energies. We attribute these to overlapping contributions from the anionic ground state of a mixed π* CO, Pt 5 d_xz_ p and the first electronically excited anionic state, which is of a σ*, Pt $d_{x^{2}-y^{2}}$ character. The overall behavior of [Pt(CO)_2_Br_2_] with respect to electron-induced dissociation is similar to what has been observed for [Pt(CO)_2_Cl_2_] [28], and the differences observed can at large be attributed to the weaker Pt−Br bond as compared to Pt−Cl and the resulting lower dissociation thresholds. Compared to the widely used cancer therapeutic cisplatin, the behavior of the Pt(II) carbonyl halides with respect to low-energy electron-induced dissociation is very different. While the dominating DEA channels in the Pt(II) carbonyl halides are the loss of one and two CO and halogen loss is negligible, the main fragments observed in DEA to [Pt(NH_3_)_2_Cl_2_] are [Pt(NH_3_)_2_Cl]^−^, Cl^−^ and [Pt(NH_3_)_2_]^−^ [20]. Hence, cleavage of the Pt−halogen bonds dominates. From the thermochemical point of view, this is not expected as the Pt−NH_3_ bond is significantly weaker than the Pt−Cl bond (1.5 eV vs. 3.3 eV in cisplatin) [20]. However, the anionic ground state of cisplatin has a repulsive σ* character unlike the π*_CO_ character of the anionic ground state of the Pt(II) carbonyl halides. Relaxation of [Pt(NH_3_)_2_Cl_2_]^−^ from its anionic ground state, through Pt−Cl bond rupture along the σ* coordinates is thus a direct process. This is not the case for the π*, CO character anionic ground state of [Pt(CO)_2_Cl_2_]^−^ and [Pt(CO)_2_Br_2_]^−^ where this process is symmetry forbidden and effective coupling of the π*_CO_ component with the respective σ* states is necessary to effectuate dissociation.

## Figures and Tables

**Table 1:** Calculated vertical excitation energy from the anionic ground state to the first anionic excited at the TDDFT wB97X-D3/ma-def2-TZVP level of theory.

Anions	Vertical Excitation SOMO+1 ← SOMO (eV)
[Pt(CO)_2_F_2_]^−^	0.58
[Pt(CO)_2_Cl_2_]^−^	0.44
[Pt(CO)_2_Br_2_]^−^	0.27
[Pt(CO)_2_I_2_]^−^	0.13

**Table 2:** Appearance energies and calculated thermochemical thresholds for all observed DEA fragments calculated at the wB97X-D3/ma-def2-TZVP and DLPNO-CCSD(T)/aug-cc-pVQZ level of theory. Threshold energies include the thermal energy of the neutral at 50 °C.

Fragments	AEs (eV)	Threshold Energy wB97X-D3/ma-def2-TZVP (eV)	Threshold Energy DLPNO-CCSD(T)/aug-cc-pVQZ (eV)
[Pt(CO)Br_2_]^−^	0.0	−2.06	−1.57
[Pt(CO)Br]^−^	0.0–2.0	−0.20	−0.017
[PtBr_2_]^−^	0.0	−0.70	−0.48
[PtBr]^−^	3.8	3.56	3.80
[Br]^−^	0.0–2.3	−0.93	−0.59

**Table 3:** Relative yields of the fragments formed by DI and DEA to Pt(CO)_2_Br_2_, average weighted CO loss per DEA and DI incident and average weighted Br loss per DI incident.

Fragments	Relative DI Contributions	Relative DEA Contributions
[Pt(CO)_2_Br_2_]^+^	1	-
[Pt(CO)Br_2_]^+/−^	0.38	1
[PtBr_2_]^+/−^	0.23	0.76
[Pt(CO)_2_Br]^+^	0.10	-
[Pt(CO)Br]^+/−^	0.07	0.02
[PtBr]^+/−^	0.25	0.004
[Pt(CO)_2_]^+^	0.01	-
[Pt(CO)]^+^	0.02	-
[PtC]^+^	0.04	-
Pt^+^	0.11	-
Br^+/−^	0.01	0.05
Average CO loss	0.7	1.4
Average Br loss	0.3	0.04

## Data Availability

The data underlying this article will be shared on reasonable request from the corresponding author.
